# Mechanistic Insights into Ag Nanoparticle Formation on β-Ag_2_WO_4_ Surfaces through Electron Beam Irradiation

**DOI:** 10.1021/acsphyschemau.4c00062

**Published:** 2024-10-31

**Authors:** André Rodrigues-Pinheiro, Amanda F. Gouveia, Elson Longo, Juan Andrés, Miguel A. San-Miguel

**Affiliations:** † Institute of Chemistry, State University of Campinas, Campinas 13083-970, Brazil; ‡ Department of Physical and Analytical Chemistry, University Jaume I, Castelló 12071, Spain; § CDMF, Federal University of São Carlos, P.O. Box 676, São Carlos 13565-905, Brazil

**Keywords:** n/p-type semiconductors, intrinsic interfaces Ag nanoparticles/β−Ag_2_WO_4_, DFT calculations, ab initio molecular dynamics simulations, metal nanoparticles, electron beam irradiation

## Abstract

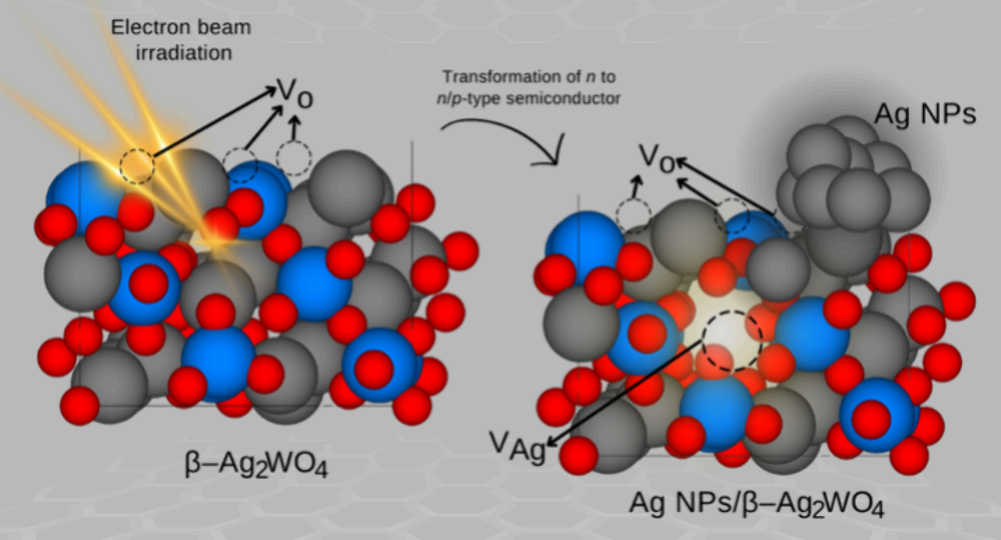

The formation of metal nanoparticles triggered by electron beam irradiations on the parent metal oxide is well-established, yet the precise mechanism remains elusive. To gain deeper insights into the time evolution of the electron beam-driven processes on (011), (111), (001), and (110) surfaces of β-Ag_2_WO_4_, we have employed density functional theory (DFT) calculations and ab initio molecular dynamics (AIMD) simulations to reveal the diffusion processes of Ag cations, the amorphization of the surfaces, and a straightforward interpretation of the time evolution for the formation of Ag nanoclusters at the β-Ag_2_WO_4_ surfaces. Present findings advanced a clear visualization, at the atomic level, of how the added electrons induce structural and electronic transformations at β-Ag_2_WO_4_ to render the formation of Ag metal nanoparticles/β-Ag_2_WO_4_ n/p-type semiconductors.

## Introduction

Advanced electron microscopy has become a well-established characterization technique and a platform for in situ nanoengineering of materials. The ability to unveil the evolution of structure and properties of nanomaterials in response to external stimuli while applying a voltage bias makes in situ transmission electron microscopy (TEM) a great advance for characterizing, producing, and manipulating nanostructures, underscoring their significant role in the nanoworld.^1−5^ This technique has been successfully utilized for revealing physical and chemical properties, enlightening details of the real-space dynamics at atomic resolution.^6^ Furthermore, electron beams are versatile tools for nanoscale fabrication processes, and several studies have investigated the growth mechanisms of metals on several metal oxide semiconductors.^7−19^ These experiments have sparked great interest in studying electron–matter interactions because the formation and growth of metal nanoparticles provoke the transformation of n-type to n/p-type semiconductors. Acquiring energetics and structural data on irradiated materials is essential to rationalize experimental observations. In particular, the microstructural evolution behaviors of single metal oxides such as TiO_2_ under electron beam irradiation have been studied.^20^

Silver tungstate Ag_2_WO_4_-based materials have attracted widespread scientific and technological interest as multifunctional semiconductors with various applications, including catalysis, photoluminescence, antibacterial agents, and gas sensors.^21−30^ This material displays three main polymorphs, α-, β-, and γ-Ag_2_WO_4_, and they have been discussed extensively in two main works.^21,31^ However, the α-Ag_2_WO_4_ (orthorhombic) and β-Ag_2_WO_4_ (hexagonal) are the most studied polymorphs with superior photocatalytic activity.^22,24,26,31−34^ One well-established strategy to enhance the activity of Ag_2_WO_4_ is to grow Ag nanoparticles (NPs) on the Ag_2_WO_4_ surface (Ag NPs/Ag_2_WO_4_). Specifically, chemical methods such as reduction by NaBH_4_^35^ or hydrazine hydrate^36^ have been employed to synthesize Ag NPs/Ag_2_WO_4_ nanostructures, increasing the methyl blue decomposition process or CO_2_ reduction under visible light, respectively. Also, Mohamed et al. developed a Ag NPs/Ag_2_WO_4_ catalyst for the methanol oxidation reaction.^37^ At the same time, Yang et al. employed this material as an efficient catalyst for reducing nitro- and azo-aromatics^38^ and could significantly enhance the corresponding performance of photocatalytic and catalytic reactions by taking advantage of the surface plasmon resonance effect.^36,38^ In a seminal work, our research group reported the spontaneous real-time observation of the sintering processes of Ag filaments on α-Ag_2_WO_4_ under high vacuum using in situ field emission scanning electron microscopy (FE-SEM).^39^ This technique uses the electron beam to induce the nucleation and growth of Ag NPs^39−47^ on α-Ag_2_WO_4_ by breaking Ag–O chemical bonds in this complex metal oxide.^48,49^ Thus, electron beam and femtosecond irradiation form Ag NPs/Ag_2_WO_4_ heterostructures, producing a potent antifungal and antitumor agent^45^ with efficient bactericidal activity.^50^ At the same time, the hybrid material chitosan/α-Ag_2_WO_4_ can make significant modifications to the material, resulting in substantial changes in its physicochemical properties. Consequently, *Escherichia coli*, *Staphylococcus aureus*, and *Candida albicans* cells are eliminated upon interaction with the irradiated composite. Additionally, we demonstrated that the interaction of this hybrid material with SARS-CoV-2 results in the inactivation of the virus through the production of reactive oxygen species (ROS).^51^ Maksoud et al.^52^ recently reported the effects of electron beam irradiation on the structural, optical, thermal, and dielectric properties of PVC/Ag_2_WO_4_ nanocomposite films. These investigations are cut examples of the golden “structure–property” relationship and have important fundamental research implications. This, in turn, is an opportunity for bringing new physics and potential technological applications.^53^

This work focuses on the fact that the β-Ag_2_WO_4_ polymorph is significantly more effective in antibacterial activity than the other phases of Ag_2_WO_4_.^31^ Previous studies have demonstrated that Ag segregation and Ag NP formation occur in β-Ag_2_WO_4_ under electron beam irradiation.^54,55^ This polymetallic oxide presents intricate structures, and the atomic-scale details of the evolution of geometry and electronic properties under electron beam irradiation of both bulk and exposed surfaces at the morphology are difficult or impossible to ascertain experimentally. Unraveling the surface atomic structures of this material is a prerequisite for a deep understanding of many surface-related applications. To overcome these issues, in this work, density functional theory (DFT) calculations and ab initio molecular dynamics (AIMD) simulations have been carried out to understand, at the atomic level, the structural and electronic rearrangements of β-Ag_2_WO_4_ surfaces as well as the formation and growth processes of Ag NPs when an electron beam is applied. We focus primarily on answering three central questions: (i) What happens with the excess electron density, simulating the electron beam, as it approaches the (011), (111), (001), and (110) surfaces of β-Ag_2_WO_4_? (ii) How are the electrons distributed in this material, and how does this electron distribution relate to atomic displacements, collective migrations, and electronic modifications? (iii) Can the quantum theory of atoms in molecules (QTAIM) provide insights into the strength of the Ag–O and W–O bonds after electron irradiation of β-Ag_2_WO_4_? The modifications induced by electron irradiation are investigated as a function of the number of added electrons (NAE) at the β-Ag_2_WO_4_ surfaces. Thereafter, AIMD simulations were performed to analyze the time evolution of geometry and electronic properties by adding electrons. The obtained results will contribute to interpreting the experimental results and provide an overall pathway and energetics of electron beam-induced processes of β-Ag_2_WO_4_ surfaces.

## Results and Discussion

### Surface Structure of the Unperturbed Systems

**Figure 1:**
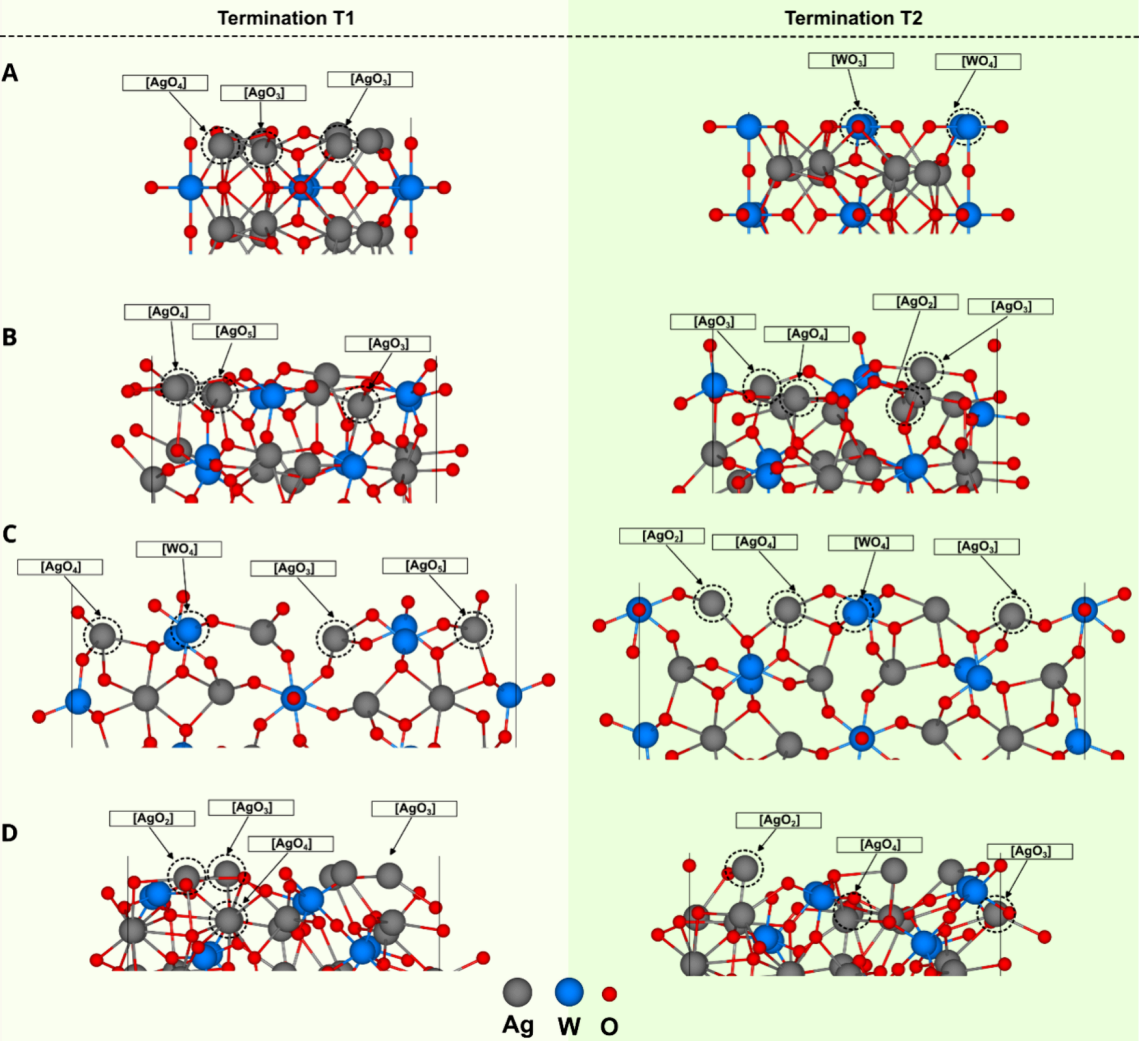
Schematic representation of the geometry of the T_1_ and T_2_ terminations of the β-Ag_2_WO_4_ surfaces: (001), (011), (110), and (111) are shown in A, B, C, and D, respectively. The gray, blue, and red spheres represent silver, tungsten, and oxygen atoms, respectively.

Figure 1B shows in detail the geometry of the predominantly exposed surfaces at the morphology of the as-synthesized samples:^55^ (110), (001), (111), and (011) surfaces with two terminations, T_1_ and T_2_. We analyzed the energetic stability of these terminations and their composition in terms of undercoordinated Ag polyhedrons. Table 1 presents the values of surface energy (γ_surf_), density of broken bonds (D_b_), the exposed Ag clusters, following the Kröger–Vink notation,^56^ and the density of Ag atoms (D_Ag_) at the surface.

**Table 1:** Values of Surface Energy (γ_surf_), Density of Broken Bonds (*D*_b_), Most Superficial Ag Clusters, and Density of Ag Atoms (*D*_Ag_) at the T_1_ and T_2_ Terminations of β-Ag_2_WO_4_

surfaces	termination	γ_surf_ (J/m^2^)	*D*_b_ (nm^–2^)	Ag clusters	*D*_Ag_ (nm^–2^)
(001)	T_1_	1.349	1.059	[AgO_4_.V_O_^*x*^], [AgO_3_.3 V_O_^*x*^], [AgO_3_.2 V_O_^*x*^]	6.5
	T_2_	1.437	0.326		0.0
(110)	T_1_	1.275	0.542	[AgO_4_.V_O_^*x*^], [AgO_3_.2 V_O_^*x*^], [AgO_3_.3 V_O_^*x*^], [AgO_2_.3 V_O_^*x*^]	5.4
	T_2_	1.633	1.288	[AgO_3_.2 V_O_^*x*^], [AgO_4_.V_O_^*x*^], [AgO_2_.4 V_O_^*x*^], [AgO_4_.2 V_O_^*x*^], [AgO_5_]	4.7
(011)	T_1_	0.383	1.148	[AgO_4_.V_O_^*x*^], [AgO_3_.2 V_O_^*x*^], [AgO_5_]	5.4
	T_2_	0.384	0.933	[AgO_2_.3 V_O_^*x*^], [AgO_4_.V_O_^*x*^], [AgO_3_.2 V_O_^*x*^]	5.4
(111)	T_1_	0.766	0.990	[AgO_3_.3 V_O_^*x*^], [AgO_2_.3 V_O_^*x*^], [AgO_4_.V_O_^*x*^], [AgO_3_.2 V_O_^*x*^]	5.5
	T_2_	0.835	1.100	[AgO_3_.2 V_O_^*x*^], [AgO_2_.4 V_O_^*x*^], [AgO_4_.V_O_^*x*^], [AgO_3_.3 V_O_^*x*^], [AgO_4_.2 V_O_^*x*^]	2.7

The γ_surf_ values reported in Table 1 increase in the following order: (011) < (111) < (110) < (001). A detailed examination of the data in Table 1 reveals the presence of oxygen vacancies and undercoordinated and distorted Ag and W clusters in all T_1_ terminations. Using the Kröger–Vink notation,^56^ the neutral oxygen vacancy (V_O_^x^) and the distorted clusters are indicated with the subscript *d*. This type of representation allows us to analyze the types of undercoordinated clusters and evaluate the density of broken bonds (*D*_b_) on each surface termination.

For β-Ag_2_WO_4_, (011) is the most stable surface. Both terminations (T_1_ and T_2_) exhibit similar stability and chemical composition on this surface. Table 1 shows that the T_1_ termination is composed of [AgO_4_·V_O_^x^], [AgO_3_·2 V_O_^x^], [AgO_3_·3 V_O_^x^], and [AgO_2_·3 V_O_^x^] clusters, while T_2_ consists of [AgO_3_·2 V_O_^x^], [AgO_4_·V_O_^x^], [AgO_2_·4 V_O_^x^], [AgO_4_·2 V_O_^x^], and [AgO_5_] clusters. Although the T_2_ termination presents a more significant number of different types of Ag clusters, it is in T_1_ that the values of *D*_Ag_ and *D*_b_ are higher.

The (111) surface at T_1_ exhibits the clusters [AgO_3_·3 V_O_^x^], [AgO_2_·3 V_O_^x^], and [AgO_4_·V_O_^x^], while at T_2_, the clusters are [AgO_3_·2 V_O_^x^], [AgO_2_·4 V_O_^x^], [AgO_4_·V_O_^x^], [AgO_3_·3 V_O_^x^], and [AgO_4_·2 V_O_^x^]. Thus, although T_2_ has fewer Ag atoms per area, these clusters are significantly less coordinated with larger values of *D*_b_.

In the (110) surface, the T_2_ termination has many broken bonds, resulting in a much more unstable termination than T_1_. In addition, the presence of more subcoordinated Ag clusters exemplifies their greater instability.

Finally, the (001) surface shows a notable difference in the composition of undercoordinated clusters. Meanwhile, T_1_, the most stable termination, comprises [AgO_4_·V_O_^x^], [AgO_3_·3 V_O_^x^], and [AgO·2 V_O_^x^] clusters, and T_2_ consists only of [WO_4_·V_O_^x^] and [WO_3_·V_O_^x^] clusters, which results in more considerable distortion and higher instability. Therefore, because the T_1_ termination is more promising regarding its energy stability than its active counterpart, all the slab models built sought to expose T_1_.

### Dynamical Effects from Ab Initio Molecular Dynamics (AIMD) Simulations

**Figure 2:**
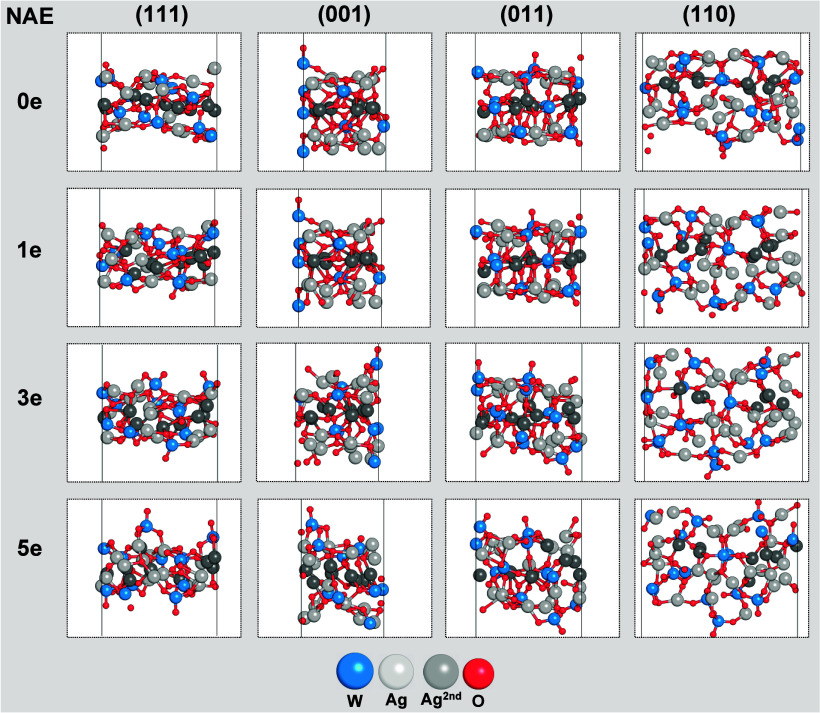
Schematic representation of the final configurations of the (111), (001), (011), and (110) surfaces at different electron additions (0, 1, 3, and 5e).

Figure 2 provides evidence of the degree of structural and electronic disorder, as well as atomistic transformations in each system upon adding electrons, which are consistent with each surface’s stability. The (111) surface retains the initial structural ordering more strongly than the other systems. In contrast, (001) displays a rapid loss of structural order due to its lower stability. This order–disorder transition on the (001) surface is accompanied by structural arrangements that induce significant changes in the local coordination of Ag cations.

Figure S1 displays the mean square displacements (MSD) calculated for the Ag atoms on the surfaces throughout the AIMD simulations. The results confirm an increase in the order–disorder transition of the (001), (011), and (110) surfaces when the NAE is 3e. The Ag atoms on the (111) facet show major deviations from their equilibrium positions. Thus, for an NAE of 1e, the MSD already indicates significant distortions compared to the neutral system. However, the loss of surface symmetry occurs only when the NAE is 5e. Therefore, at low values of NAE, the (001), (011), and (110) surfaces exhibit faster and more substantial amorphization than the (111) surface.

To gain insights into the structural modifications, we calculate the pair correlation function, *g*(*r*), and the results for the bulk and surfaces of β-Ag_2_WO_4_ are shown in Figures S2, S3, and 3. The most distinct peaks in the bulk correspond to the first, second, third, and fourth Ag–Ag distances. However, for the surfaces, an abrupt change occurs where the maximum peak of the first distance increases, while the second and third peaks disappear. It should be noted that the first peak shifts to shorter Ag–Ag distances, while its intensity decreases when the NAE increases. This behavior indicates that Ag maintains its preferred local coordination, while the broadening of the peaks reflects an intensification of the local disorder of the structure. Nevertheless, these disordered surfaces possess some degree of short-range order, which may be related to local changes in bond lengths between neighbors associated with coordination environments distinct from those of Ag. The addition of electrons disrupts the ground state equilibrium between atoms and electrons, thus producing mechanical stresses and forces that result in a significant degree of structural disorder.

**Figure 3:**
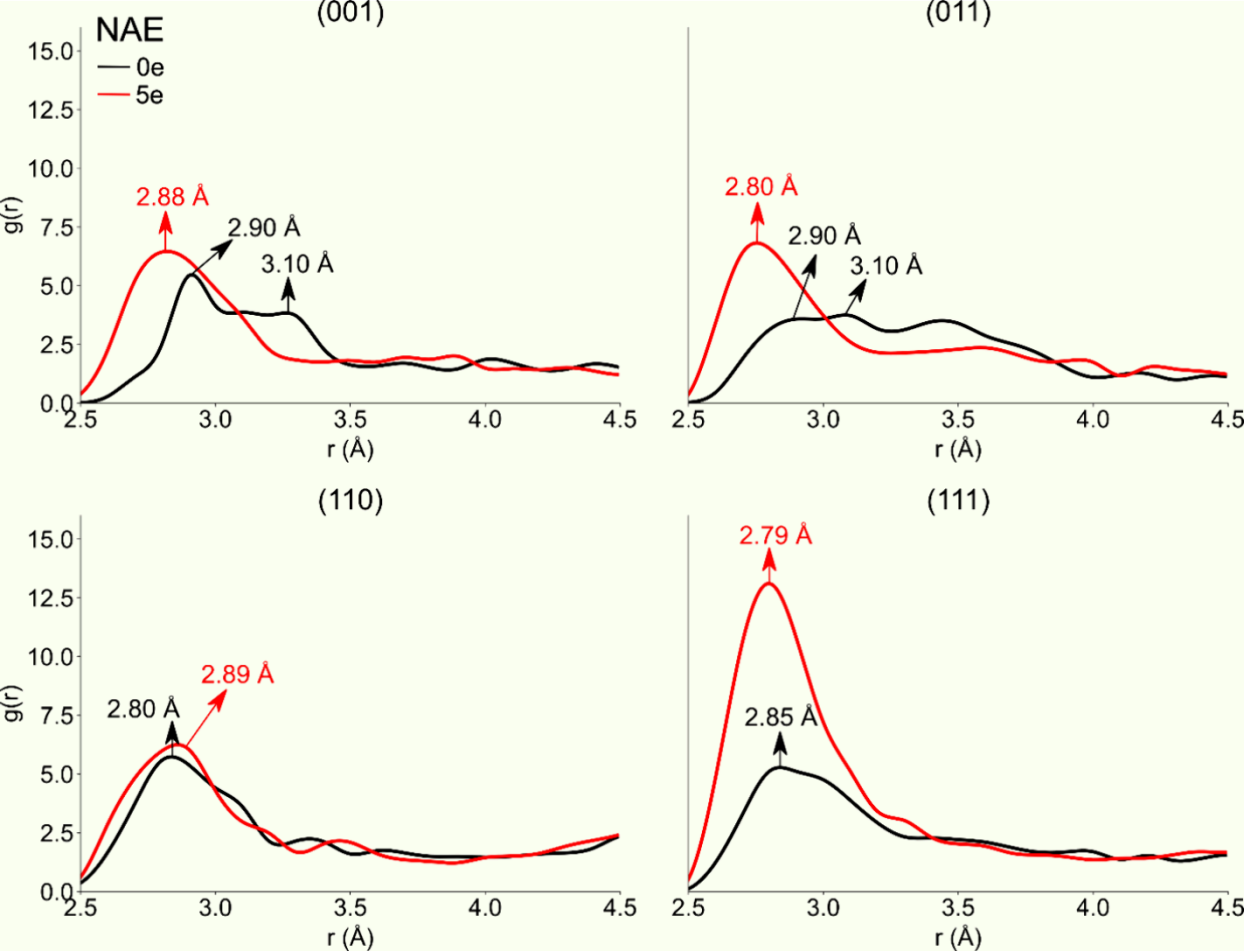
Pair correlation function of the Ag–Ag distances for the β-Ag_2_WO_4_ surfaces. The lines in black and red represent the surfaces with NAE 0e and 5e, respectively.

Specifically, the (001) surface (Figure 3) shows that the Ag–Ag distances in the Ag clusters are in the range 3.03–3.20 Å, which are already shorter than those in the Ag bulk (3.20 Å). According to Chen et al.,^57^ the Ag–Ag bond length for small clusters is between 2.85 and 2.90 Å. As the NAE increases, the Ag–Ag distances are further reduced to 2.88 Å. In this context, the clusters formed in the interior of the surfaces exhibit distances similar to those observed in small Ag clusters (Figure S3).

A similar effect occurs in the (011) surface (Figure 3), where the disappearance of the three most prominent peaks and their subsequent merge at 2.81 Å can be observed. On the other surfaces, (110) and (111), it is evident that the Ag cations are at shorter distances than in the bulk; a more significant observation at (111) (Figure 3) with the broadening of the peak at 2.85 Å indicates that more Ag cations are approaching. In the case of (110), few structural changes are observed. Similarly, we also analyzed the *g*(*r*) plots for Ag–O distances (Figure S1). Thus, it becomes evident that as the NAE increases, the first peaks tend to unify and shift to larger distances, reflecting elongation of the Ag–O bonds in the [AgO_*x*_] clusters.

Ag–Ag distances in the most representative surface configurations at varying added electrons were analyzed to confirm the structural features. The results indicate that increased NAE forms dimers and internal clusters on some surfaces. These areas feature the creation of Ag vacancies, transforming the material from n-type to n/p-type semiconductors in local regions. Consequently, electron irradiation produces a new semiconductor with a random distribution of Ag and oxygen vacancies.

**Figure 4:**
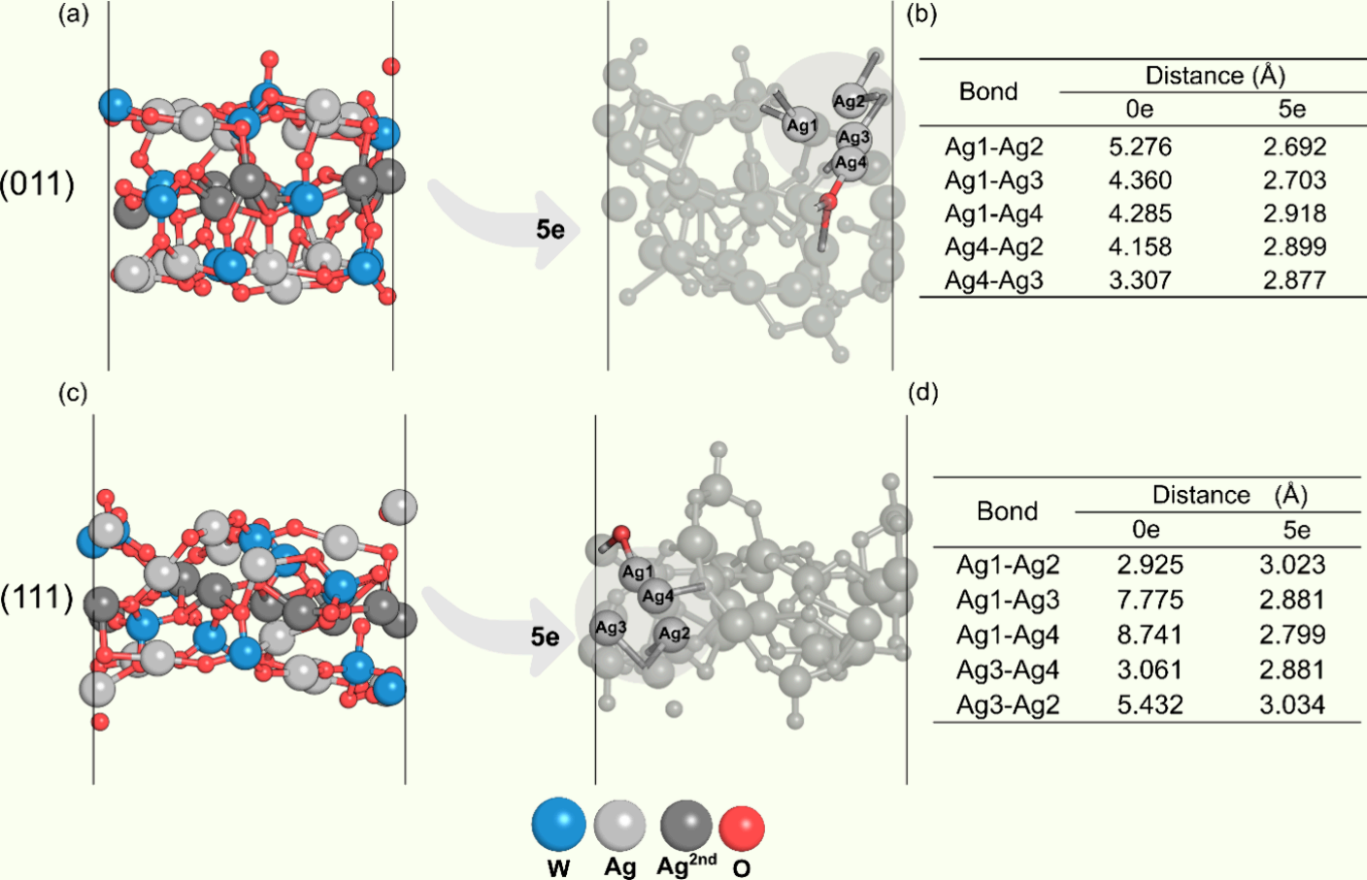
Representative surface configurations of the (011) and (111) surfaces for NAE of 0 and 5e in (a,c), respectively. In (b,d), the Ag–Ag distances of the tetrahedron formed internally on the surface are shown.

An analysis of the average Ag–Ag distances between Ag atoms of the tetrahedrons formed on the (011) surface (Figure 4b) shows that these distances are longer than 4.0 Å for the neutral system. As NAE increases, this distance tends to fall to 2.80 Å, allowing the formation of a Ag cluster. The (001) surface, as well as (011), shows the formation of an internal Ag cluster (Figure 4c). When analyzing the average distances between Ag atoms in the formed cluster, a significant reduction in Ag–Ag distances is observed, going from 3.73 to 2.90 Å.

The (111) surface exhibits significant changes in the Ag–Ag distances. Figure 4 illustrates the formation of a Ag tetrahedron within the surface. An analysis of the average distances between the atoms that compose this cluster reveals that under neutral conditions and when adding 5e, the average distances are 4.88 and 2.85 Å, respectively. These distances closely resemble those observed in the cluster found on the (011) surface, suggesting that electron addition induces the formation of Ag clusters. Notably, Ag clusters form most prominently on the (011), (111), and (001) surfaces. On the contrary, no Ag cluster was observed internally on the (110) surface.

These transformations can be analyzed from the relative potential energies shown in Figures S4 and S7, which demonstrate an increase in potential energy associated with the loss of structural order induced by electron absorption. This increase in energy occurs on all surfaces, implying that all surfaces become amorphous. The difference is that on the (011), (110), and (001) surfaces, it is necessary to introduce 5e to observe diffusion and the subsequent formation of metallic Ag agglomerates. In contrast, on the (111) surface, only 3e is sufficient to produce the deformations that culminate in the agglomeration of Ag atoms.

These results closely resemble the subvalent compounds reported in the literature,^58,59^ where the phenomenon of electron excess is discussed. Similar to those systems, we observe an electron excess in the isolated silver clusters, which exhibit metallic characteristics akin to bulk silver. In the systems described here, however, the electron excess is influenced by the electron irradiation from the microscope, resulting in electron dosages higher than those observed in subvalent compounds. Consequently, the appearance of metallic substructures leads to the formation of intriguing systems with potential novel applications that warrant further exploration in future research.

### Electronic Effects

**Figure 5:**
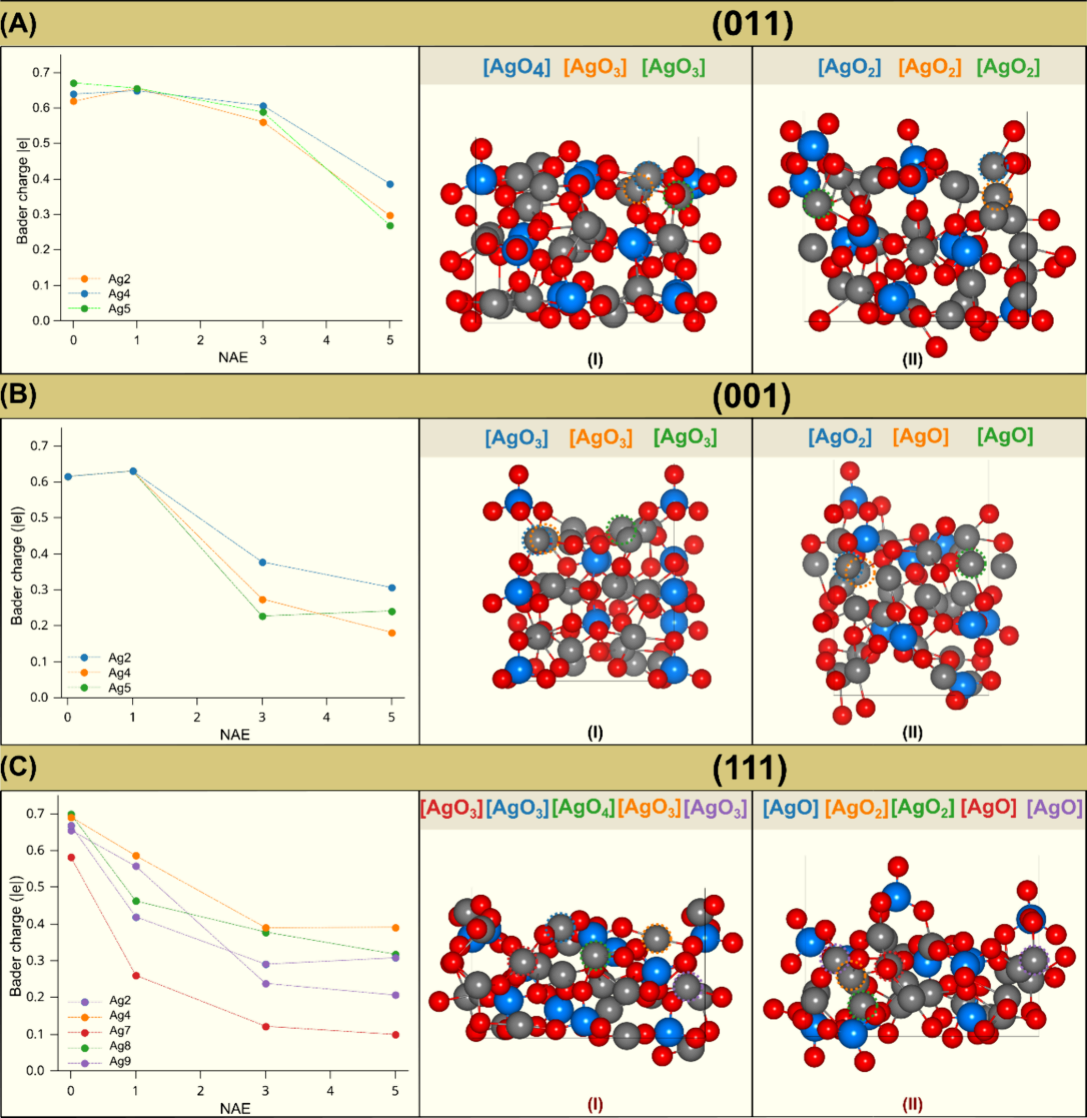
Bader charges of the most superficial Ag atoms for surfaces (A) (011), (B) (001), and (C) (111). The graphs on the left depict the variation in Bader charges as a function of the number of absorbed electrons (NAE) for different silver atoms (Ag2, Ag4, Ag5, Ag7, Ag8, and Ag9). On the right, surface models are shown for (I) the initial state (NAE = 0) and (II) after the absorption of 5 electrons (NAE = 5e). The structural representations emphasize the various Ag coordination polyhedrons, such as [AgO_4_], [AgO_3_], and [AgO_2_], which are color-coded consistently with the corresponding atoms in the graphs on the left.

To understand each system’s electron density distribution, we analyzed each surface’s representative configuration and the internally formed clusters. Figure 5 shows the effective Bader charge of the most superficial Ag cations as a function of the NAE.

An analysis of the results shows that the values of the Bader charges at the Ag atoms decrease as NAE increases. This reduction is more significant on (111) and (001) surfaces, where for higher NAEs, the average charge is approximately +0.30 |e|. On the other surfaces, the average Ag charge for a higher NAE is +0.50 |e|, indicating that the different chemical environments of the surfaces influence electron absorption. The (011) and (110) surfaces also exhibit more Ag cations with similar effective charges. Therefore, this difference in the electronic environment of each surface suggests that the effects of electron absorption are more significant on (111) and (001) surfaces than on the (011) and (110) surfaces.

On the (111) surface, a tendency of reduction of Ag atoms in the less coordinated polyhedrons, namely, [AgO_2_] and [AgO], is observed. On the other hand, more coordinated polyhedrons do not exhibit a significant reduction. A similar effect is also observed in the (001) surface, mainly in the [AgO] clusters. This behavior is interesting, as it indicates the existence of distinct mechanisms in the formation of metallic Ag. One of them is associated with the most coordinated clusters, which, in turn, require more electrons than those of the less coordinated clusters. The other mechanism is related to less coordinated polyhedrons, as they have a greater tendency to incorporate electrons. Extending this analysis to the Ag cations at the clusters of the (011) and (111) surfaces, it can be seen that the average Bader charge was reduced.

**Figure 6:**
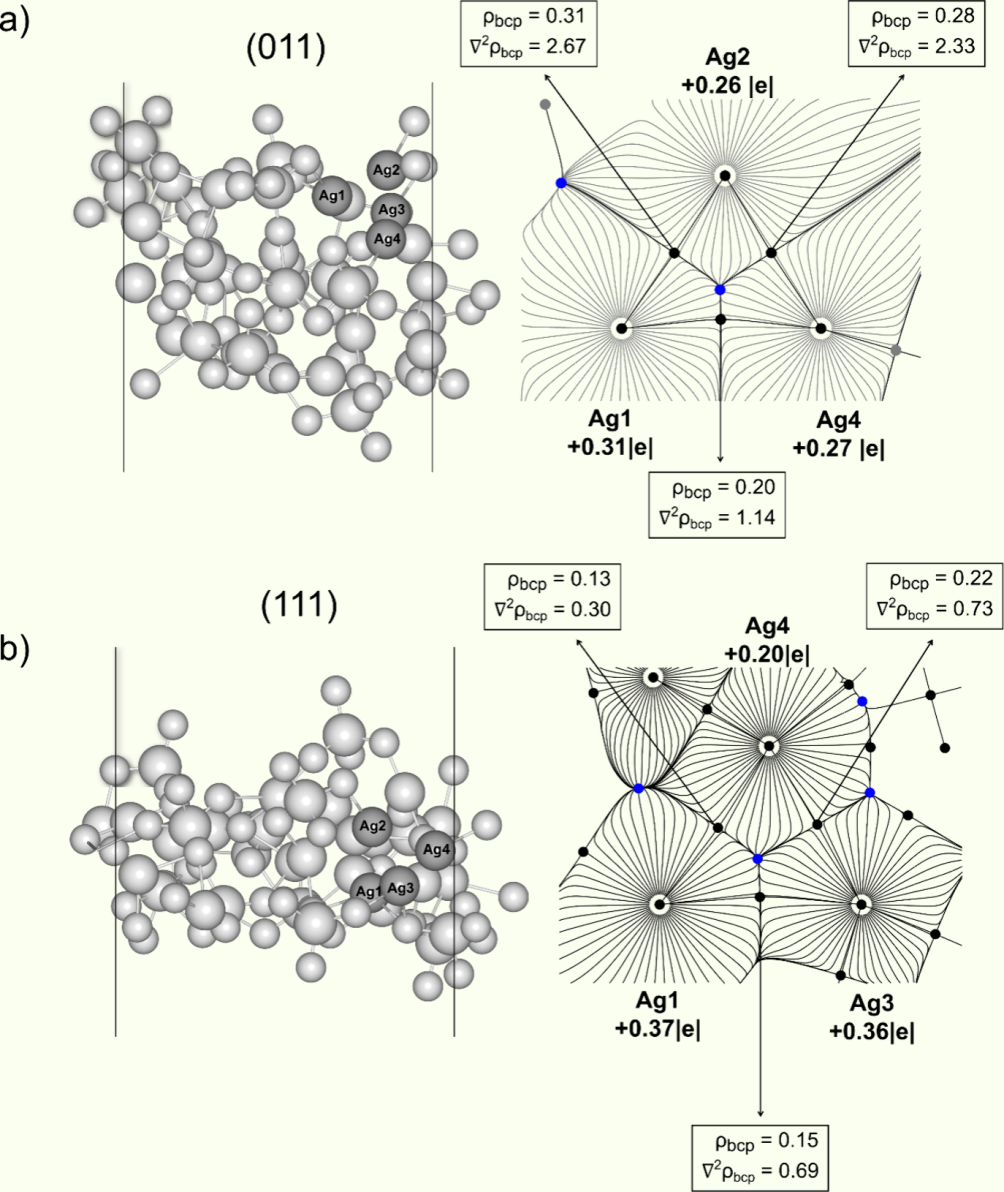
Representation of the clusters formed within the (011) and (111) surfaces in (a) and (b), respectively. The Bader charges were included for the silver atoms constituting these internal clusters as well as the associated topological parameters, such as Laplacian (∇^2^ρ) and charge density (ρ).

Figure 6 shows the Bader charge values for the Ag atoms forming the internal Ag clusters in the (011) and (111) surfaces. All Ag atoms significantly reduced their effective charge when adding 5e. Specifically, for the (011) surface, the mean Ag charges are reduced from +0.67|e| to +0.28|e|; however, for (111), the charge reduction is more substantial for some Ag cations.

The properties related to the charge density (ρ) at the bond critical points (BCP) (3,–1), as well as their Laplacian (∇^2^ρ), for the Ag–Ag distances in the clusters formed inside the (011) and (111) surfaces, are shown in Figure 6, Tables S2 and S3. As can be seen, adding electrons to the surfaces produces a Ag cluster with distances and topological parameters equivalent to metallic Ag. The topological properties at the last electron addition indicate that Ag–Ag bonds are formed during the electron injections. Although metallic clusters are formed inside the surfaces, there is a difference in the topological parameters for the (011) and (111) surfaces. According to these results, the internal clusters of the (011) surface show stronger interactions than those of the (111) surface. This indicates that the approximations between Ag atoms reported earlier are more likely to be formed in (011) due to the weaker interactions.

Therefore, these theoretical results shed light on the Ag cluster formation induced by electron irradiation on the β–Ag_2_WO_4_ surfaces. This process is evident for all systems, but the effect is more pronounced on the (111) and (011) surfaces compared to the (110) and (001) surfaces. Even on the latter two, the formation of metal clusters occurs, starting with dimers and trimers and subsequently forming larger Ag nanostructures, eventually leading to filaments similar to those observed in experimental studies reported by our research group.^54,55^

β-Ag_2_WO_4_ is an intrinsic n-type semiconductor due to oxygen vacancies, which make electrons the primary charge carriers. The absorption of additional electrons in random regions induces the diffusion of Ag atoms, leading to the formation of metallic Ag clusters, creating silver vacancies, and consequently generating local p-type regions. Thus, this theoretical analysis characterizes a novel n/p-type semiconductor composed of an n-type semiconductor matrix with a random distribution of localized p-type regions.

## Conclusions

In summary, the present DFT calculations and AIMD simulations unveil how the (011), (111), (001), and (110) surfaces of β-Ag_2_WO_4_ are modified when an electron beam is applied. We evaluate the changes in structure and electronic properties and extend the fundamental understanding, at the atomic level, of the underlying mechanism of electron beam-driven processes. Our results offer a straightforward interpretation of the time evolution as a function of added electrons, wherein the (011) and (111) surfaces present a significantly higher propensity to generate Ag nanoparticles than the (001) and (110) surfaces. Overall, the present work provides fine details of surface patterning to provide the mechanism of Ag NP formation and evolution at β-Ag_2_WO_4_ to render an n/p-type semiconductor.

## Computational Methods

Total energy DFT-based calculations were performed using the *Vienna Ab Initio Simulation Package* (VASP)^60,61^ version 5.4.4. The Kohn–Sham equations were solved using the generalized gradient approximation (GGA), specifically with the Perdew–Burke–Ernzerhof (PBE) functional with Grimme D3 corrections.^62,63^ A plane wave basis set was employed to describe the valence electrons, and the projector augmented wave (PAW) method was used for the interaction of nuclei with valence electrons (4d and 5s for Ag atoms, 5d and 6s for W and 2s, and 2p atoms for O atoms).^64^ A plane wave cutting energy of 550 eV was employed to obtain wholly minimized structures. The conjugate gradient method was used to converge the structures until the Hellmann–Feynman forces were in the order of 0.001 eV × Å^–1^ per atom.

The first Brillouin zone was integrated with converged Monkhorst–Pack grids,^65^ a 9 × 9 × 9 grid for the bulk and a 3 × 3 × 1 grid for the surfaces. However, the AIMD simulations used only the gamma point to reduce the computational time. These grids have been tested to be precise enough and also adopted for other similar systems in our group.

**Figure 7:**
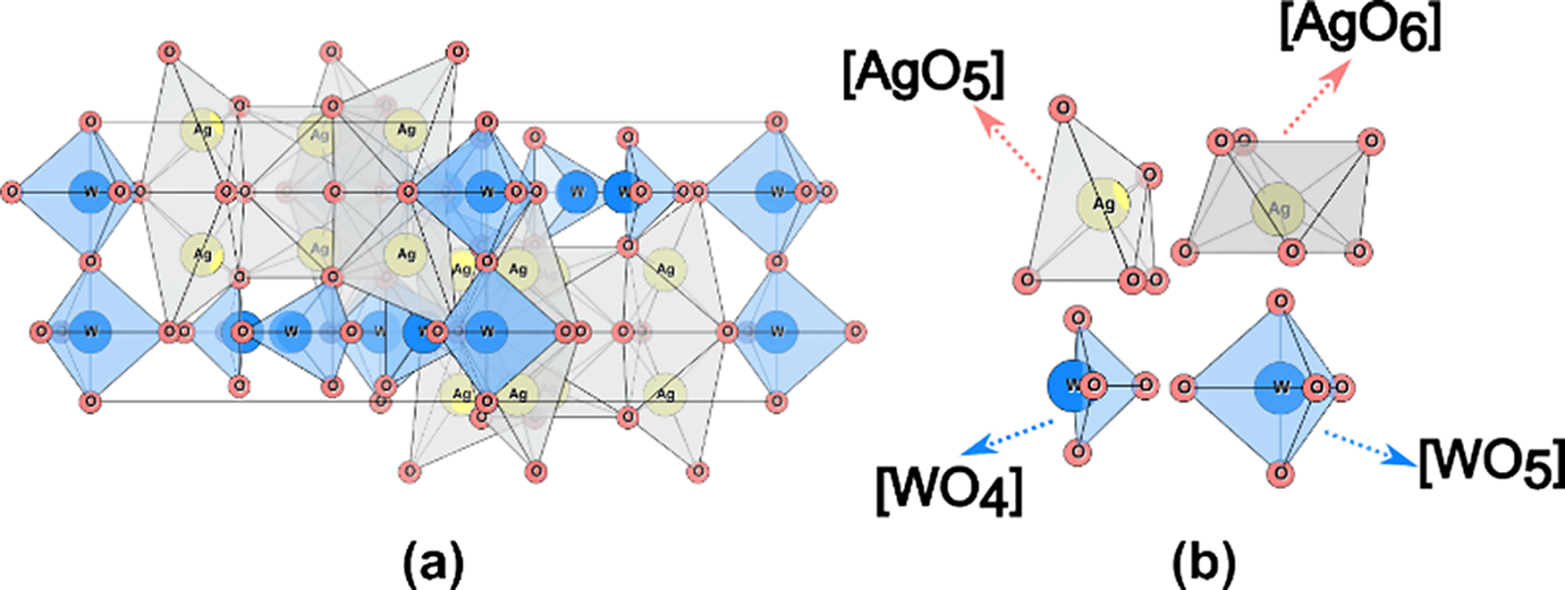
Representation of (a) β-Ag_2_WO_4_ bulk structure and (b) building polyhedrons.

The β-Ag_2_WO_4_ bulk structure consists of four different types of clusters [AgOx] (*x* = 5 and 6) and [WOy] (*y* = 4 and 5). These clusters form a hexagonal lattice (space group P63m) with eight formula units (*Z* = 8) per cell, as illustrated in Figure 7. The optimized lattice parameters were calculated to be *a* = *b* = 11.07 Å, *c* = 7.68 Å, in agreement with experimental values.^55^

The surface energy (γ_surf_) for each T_1_ and T_2_ termination was evaluated following eq 1.^66,67^
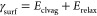
1

The cleavage energy, *E*_clvag_, was calculated using eq 2 from the two terminations, T_1_ and T_2_, due to the asymmetric character of the surface model. *E*_bulk_ and *E*_slab_^unrelax^ represent the total energies for the “bulk” and the nonrelaxed slab models, respectively; *n* is the ratio between the number of molecules in the “slab” and “bulk”; and *A* refers to the surface area.
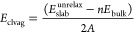
2

Then, the T_1_ and T_2_ terminations were relaxed separately. The surface energy related to this process was calculated using the eq 3
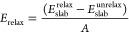
3where *A* represents the surface area for T_1_ or T_2_ termination.

It is possible to estimate the energy required to create the surface model from the set of obtained energies by considering the number of broken bonds and the changes in the coordination numbers of the surface cations. Furthermore, the density of broken bonds (*D*_b_) can be calculated as follows:
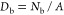
4where *N*_b_ represents the total number of broken bonds in the surface area (A).^68^

Atomic charges were estimated using the QTAIM.^69^ This approach is an essential tool for analyzing electronic density and chemical bonds as it quantifies bonding forces between atoms by identifying bond critical points (BCP) and bond paths. Critical points are found through zero-value points in the first-order derivatives of the electron density function. These points can be classified according to nine possible variations of the second-order derivatives, and by mapping these points, it is possible to identify chemical bonds.^70,71^ The second-order derivative, the Laplacian (∇^2^ρ), is essential as it indicates the electron density concentration along the bond path between two atoms, implying a shared interaction. Furthermore, shared interactions are characterized by ∇^2^ρ < 0 and electron density greater than 0.1 au. On the other hand, when the values are ∇^2^ρ > 0, the interaction is classified as a closed-shell bond.^72^

The atomic charges were obtained by integrating the electron density in atomic basins, Ω, and subtracting the nuclear charge, *Z*, of the central atom of the polyhedron, according to eq 5:
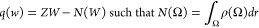
5

The (001), (011), (111), and (110) surfaces were built using the bulk structure obtained in a previous study.^55^ Each surface model was constructed from a layer with 12 molecular units of β-Ag_2_WO_4_, which were completely relaxed and employed as the initial configurations in the AIMD simulations. The ionic and electronic convergence criteria were 0.01 eV/Å for the Hellmann–Feynman forces and 1 × 10^–5^ eV for electronic convergence, respectively.

From a set of six terminations for the (011), (111), and (110) surfaces, the most stable terminations were determined, while the (001) surface had four different terminations. The cleavage energy values for the constructed surfaces are presented in Table S1 in the Supporting Information (SI). The termination with the lowest cleavage energy was selected for each surface. The density of Ag atoms (*D*_Ag_) was calculated based on the total amount of Ag atoms exposed per unit area, as defined by eq 6:
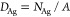
6where *N*_Ag_ represents the total number of exposed Ag atoms, and *A* denotes the surface area unit.^68^

The electron irradiation was simulated by adding electrons to the surfaces with the NELECT flag, as implemented in the VASP code. The AIMD simulations were performed by using the NVT ensemble and the Nose–Hoover thermostat. The protocol employed was as follows: initially, each surface was thermally equilibrated at 300 K for 2 ps to ensure the system’s thermal equilibrium. At each step, systems with a number of added electrons (NAE) of 0, 1, 3, and 5 were considered for each surface, and the structural evolution was monitored to identify the most representative configurations. This method was successfully employed to elucidate the metal Ag cluster formation in Ag_3_PO_4_ when adding electrons.^73,74^

## Supporting Information Available

Description of the cleavage energy of the possible terminations of the β-Ag_2_WO_4_ surfaces; topological parameters of the clusters formed within the surfaces; data related to MSD and pair correlation functions of the Ag–O distances and the bulk material; potential energy profile as a function of simulation time; and validation of the surface models (PDF)
