# Supplementary material for: Mechanistic Insights into Ag Nanoparticle Formation on β-Ag2WO4 Surfaces through Electron Beam Irradiation
Source: ACS Phys Chem Au. 2024 Oct 31;5(2):139–50. doi: 10.1021/acsphyschemau.4c00062 (PMC11950856; doi:10.1021/acsphyschemau.4c00062)
Supplement: Supplementary file 1 — pg4c00062_si_001.pdf [file pg4c00062_si_001.pdf]

Mechanistic Insights into Ag Nanoparticle Formation on β-Ag_2WO_4 Surfaces through Electron Beam Irradiation

André Rodrigues-Pinheiro[a], Amanda F. Gouveia[b], Elson Longo[c], Juan Andrés[b], Miguel A. San-Miguel[*c]

a. Institute of Chemistry, State University of Campinas, Campinas 13083-970, Brazil
b. Department of Physical and Analytical Chemistry, University of Jaume I, Castello 12071, Spain
c. CDMF, Federal University of São Carlos, P.O. Box 676, São Carlos 13565-905, Brazil

[*]Email: smiguel@unicamp.br

Supplementary Information

Topological electron density and geometrical parameters

Table S1. Cleavage energy of all terminations for the (011), (001), (111), and (110) surfaces.

	Surfaces			
Terminations	(011)	(001)	(111)	(110)
1	0.743	1.190	2.359	2.033
2	1.525	1.803	1.909	3.143
3	3.247	1.807	2.434	1.451
4	2.583	1.808	3.016	1.972
5	2.763	--	2.018	3.124
6	1.881	--	2.291	2.417

Table S2. Values of the $d_{Ag\text{-}Ag}$ (Å) distances and topological parameters ($\boldsymbol{\rho}(\mathbf{r})$ and $\nabla^2\boldsymbol{\rho}(\mathbf{r})$) of the cluster formed on the (011) surface under a NAE of 5e. The electron density and the Laplacian of the electron density of bulk FCC Ag are included for comparison.

System	$d_{Ag\text{-}Ag}$ (Å)	$\boldsymbol{\rho}(\mathbf{r})$	$\nabla^2\boldsymbol{\rho}(\mathbf{r})$
Ag cluster	2.91	0.21	1.32
	2.87	0.22	1.54
	2.89	0.21	1.49
	2.70	0.31	2.48
	2.69	0.31	2.59
	2.66	0.32	2.79
Metallic Ag	2.87	0.21	1.59

Table S3. Values of the $d_{Ag\text{-}Ag}$ (Å) distances and topological parameters ($\boldsymbol{\rho}(\mathbf{r})$ and $\nabla^2\boldsymbol{\rho}(\mathbf{r})$) of the cluster formed on the (111) surface under a NAE of 5e. The electron density and the Laplacian of the electron density of bulk FCC Ag are included for comparison.

System	$d_{Ag\text{-}Ag}$ (Å)	$\boldsymbol{\rho}(\mathbf{r})$	$\nabla^2\boldsymbol{\rho}(\mathbf{r})$
Ag cluster	2.99	0.17	1.20
	2.97	0.16	1.22
	2.80	0.23	1.96
	2.96	0.18	1.28
Metallic Ag	2.87	0.21	1.59

Mean Square Displacements

The MSD indicates the deviation of the position of Ag cations relative to a reference position over time.

$$MSD(m) = \frac{1}{N_{particles}} \sum_{i=1}^{N_{particles}} \frac{1}{N-m} \sum_{k=0}^{N-m-1} (r_i(k+m) - r_i(k))^2$$

Here, r_i represents the position of the atom during the simulation, while $N_{particles}$ and N represent the number of Ag cations and the total number of frames, respectively. Based on this definition, the MSD was calculated using the displacement of Ag cations across all windows of length m. As referenced, our study employed the Fast Fourier Transform (FFT) algorithm.[2,3] The MSD values for each NAE on all studied surfaces are illustrated in Figure S1.

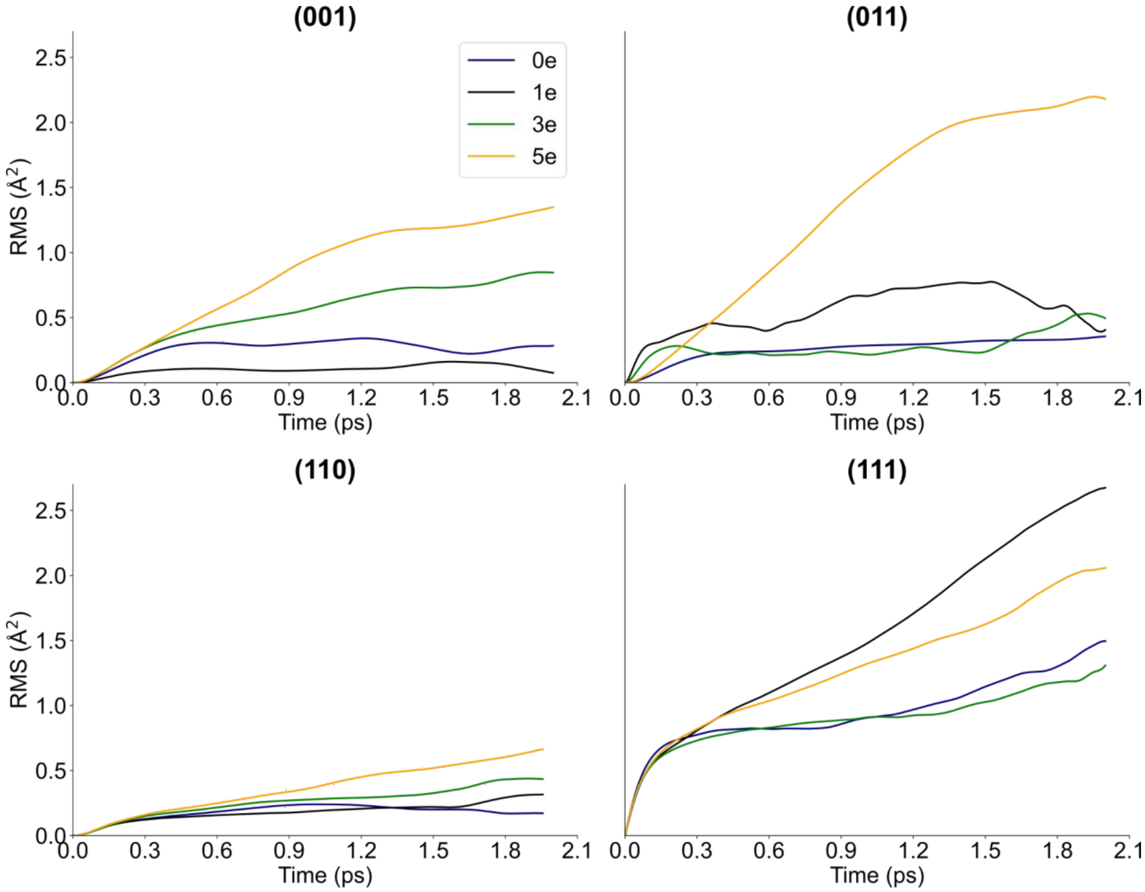

Figure S1. Mean square displacement for the surfaces (001), (011), (110), and (111) throughout the AIMD simulations over different values of NAE.

Pair Correlation Function

The position of a specific atom can be estimated using the pair correlation function[1], which measures the distance of an atom *i* (r_i) relative to another atom *j* at a distance r_{ij}. Thus, the function that enables the calculation of the distances of Ag ions can be defined as follows:

$$g(r) = \frac{1}{4\pi r^2}\frac{1}{N}\sum_{i}\sum_{j\neq i}\langle\delta(r_i - r_{ij})\rangle$$

Where N is the number of Ag atoms present on each surface. The g(r) functions for all β–Ag_2WO_4 surfaces are shown in Figures 3, present in the main text, and S2 and S3 of this supplementary information.

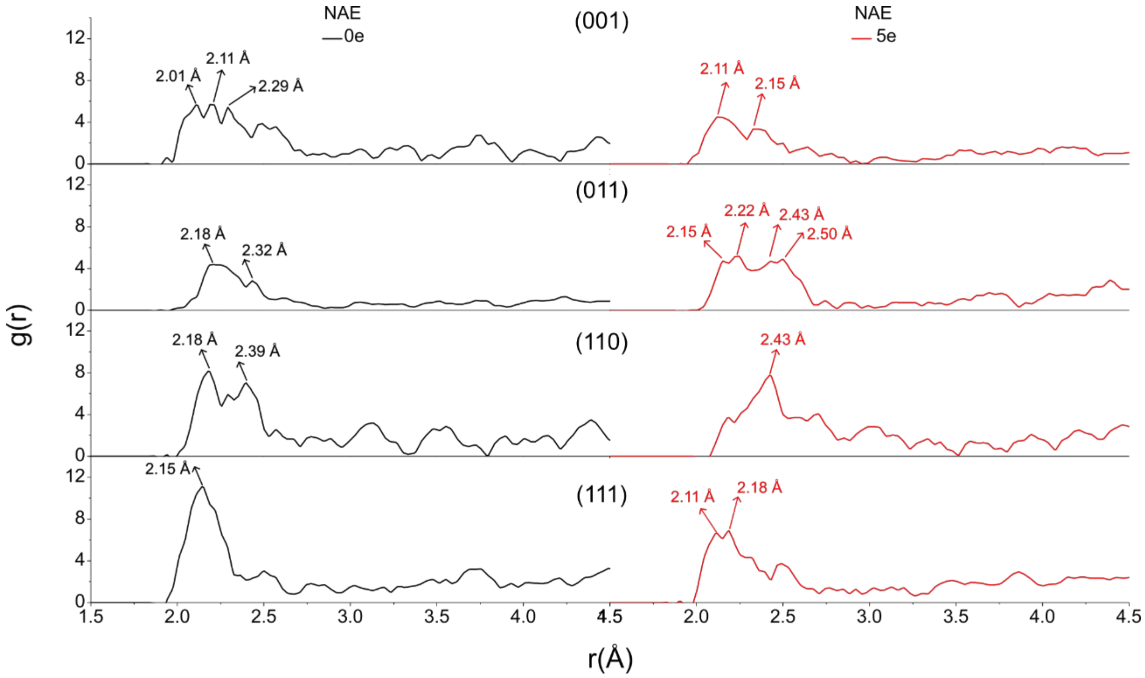

Figure S2. Pair correlation functions g(r) of Ag–O bonds in the (001), (011), (110), and (111) surfaces when 0 and 5e are added, respectively. The black and red lines correspond to NAE 0 and 5e.

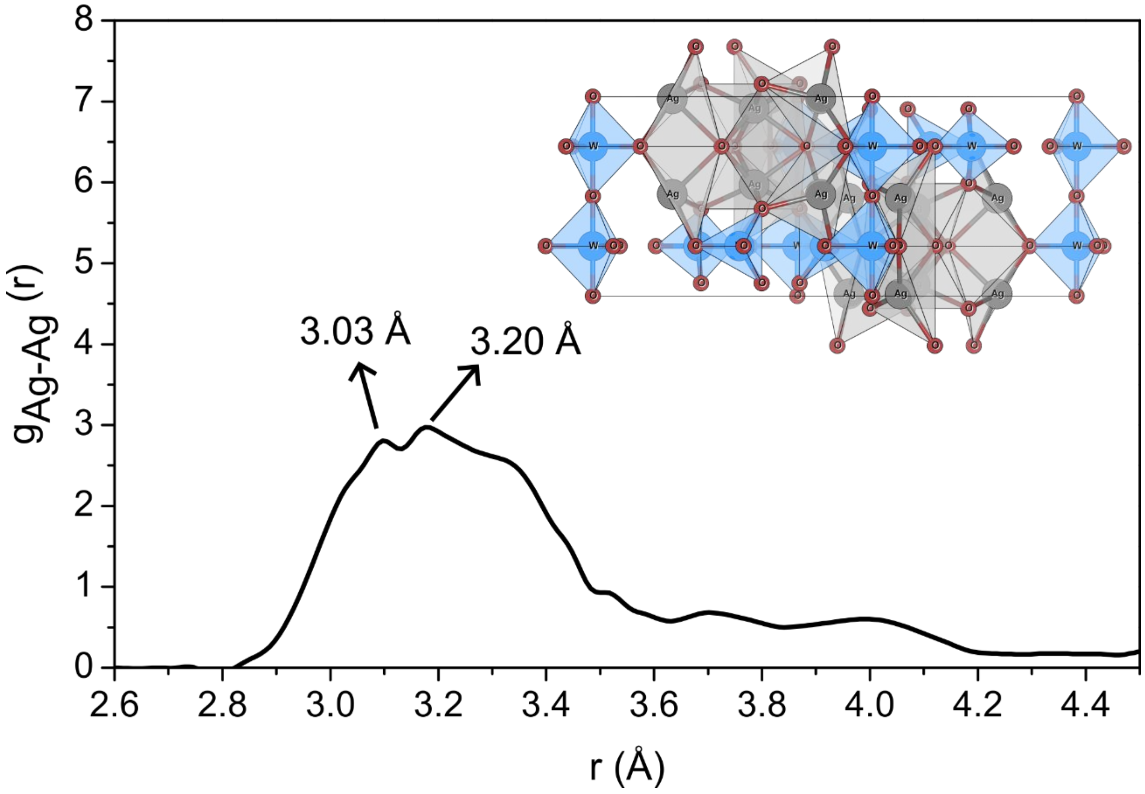

Figure S3. The pair correlation function of the Ag-Ag distances of the β–Ag_2WO_4 bulk.

Potential energy profile for surface modification

Figures S4 to S7 show potential energy profiles as a function of the simulation time during which the surface order is significantly changed.

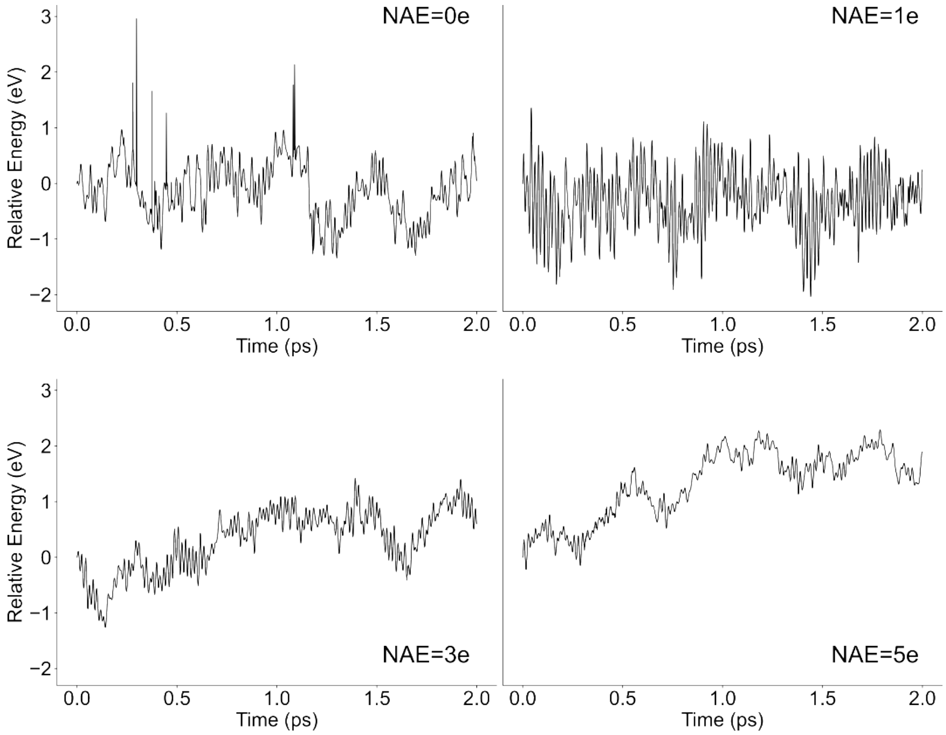

Figure S4. Energy profile for the (001) surface as a function of time simulation.

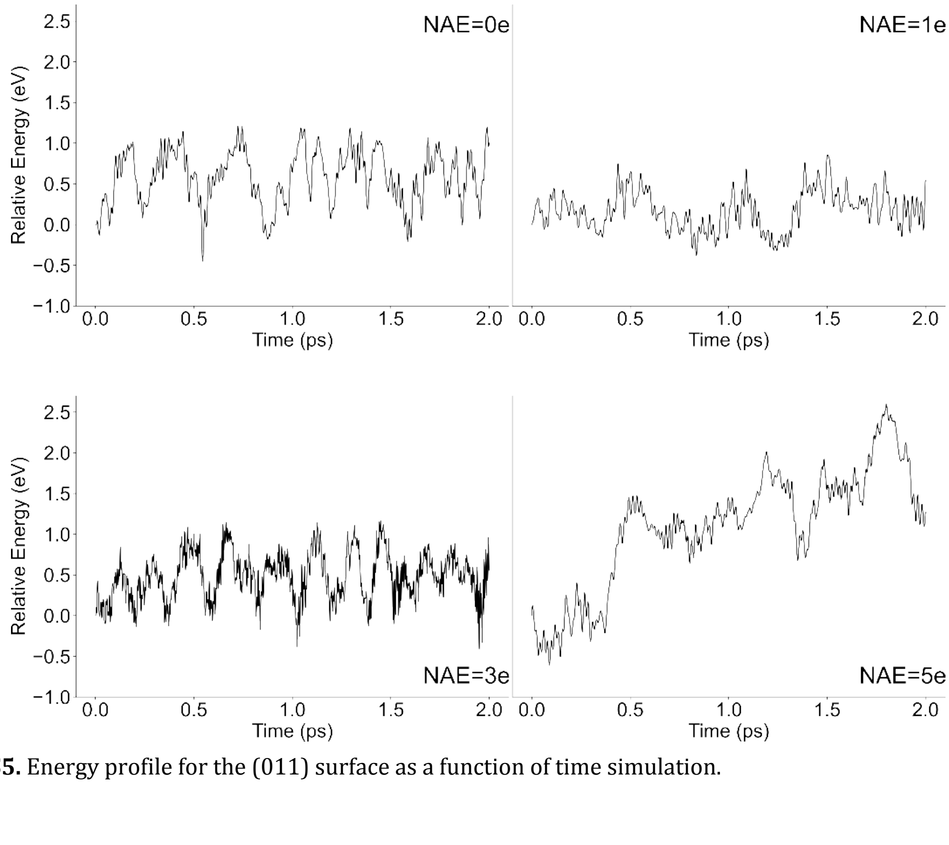

Figure S5. Energy profile for the (011) surface as a function of time simulation.

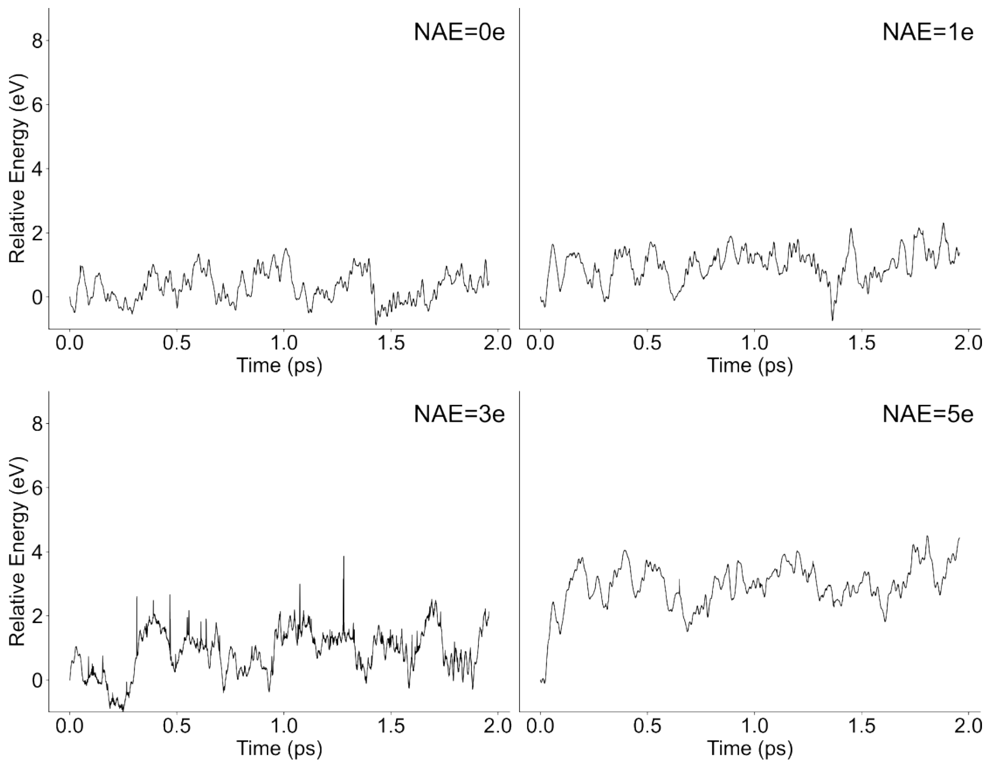

Figure S6. Energy profile for the (110) surface as a function of time simulation.

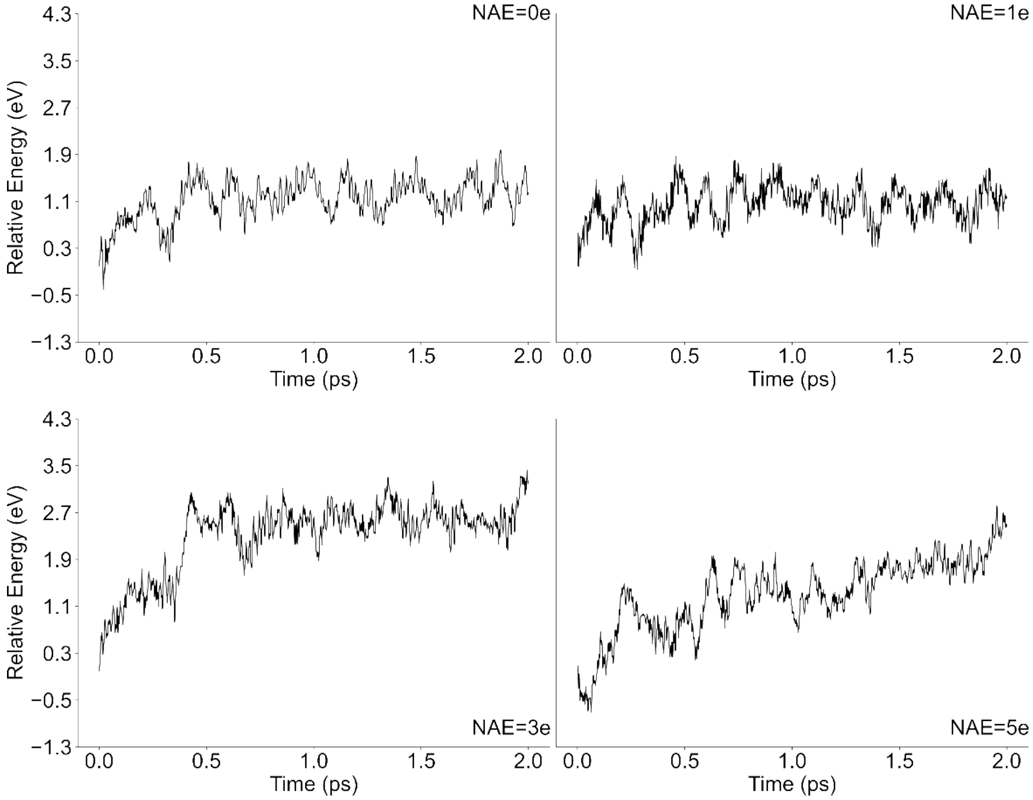

Figure S7. Energy profile for the (111) surface as a function of time simulation.

Validation of the surface models

The constructed surface models were characterized by containing 84 atoms ($Ag_{24}W_{12}O_{48}$) in each of the studied directions, namely, [001], [011], [110], and [111]. To produce each surface, we cut the Ag_2WO_4 bulk along the cited directions to construct 2D slab models with a 15 Å vacuum layer along the c-axis. A dipole correction was employed to correct the electrostatic effects arising from the asymmetric terminations of each surface model.

Initially, we determined the optimal slab thickness by performing static calculations centered at the Γ point for various slab sizes. We found that, for all surfaces when there is a slab with 12 molecular units ($Ag_{24}W_{12}O_{48}$), the variation in total energy is less than 0.05 eV/atom.

Additionally, a 2×2 model was studied to verify if the effects associated with forming metallic clusters observed within the (011) and (111) surfaces did not result from a reduced area model. To this end, we constructed the (011) surface containing ($Ag_{96}W_{48}O_{192}$) with an area of 597 Å^2, and a vacuum of 20 Å was added along the c-axis.

The effects associated with the increase in NAE are observed in the transformations that occur throughout the entire surface lattice and in the loss of structural order of the Ag atoms (Figure S8a-b). Due to the transformations associated with atomic processes, the Ag cations exhibit significant changes in their local coordination, reducing the distances between them.

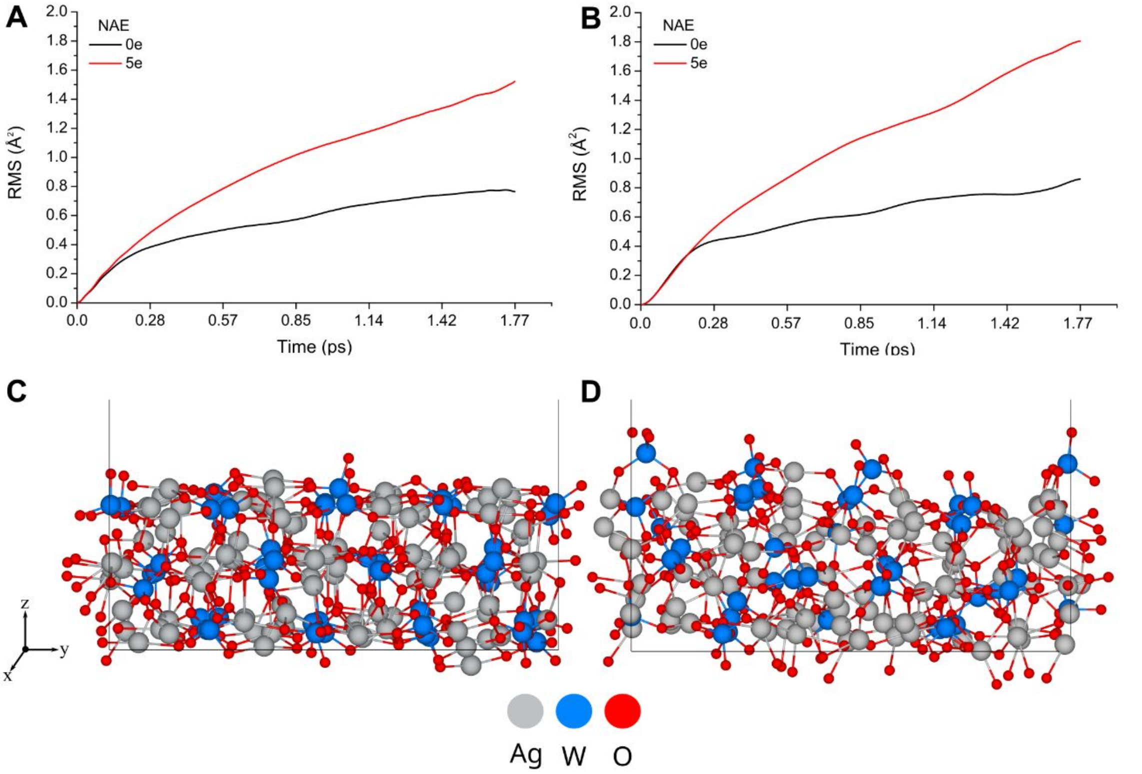

Figure S8. Representation of the 2×2 supercell of the (011) surface of β-Ag_2WO_4 ($Ag_{96}W_{48}O_{192}$). Panels A and B illustrate the MSD of all the atoms in the surface lattice and only the Ag cations, respectively. Panels C and D represent the (011) surface when the applied NAE is 0 and 5e, respectively.

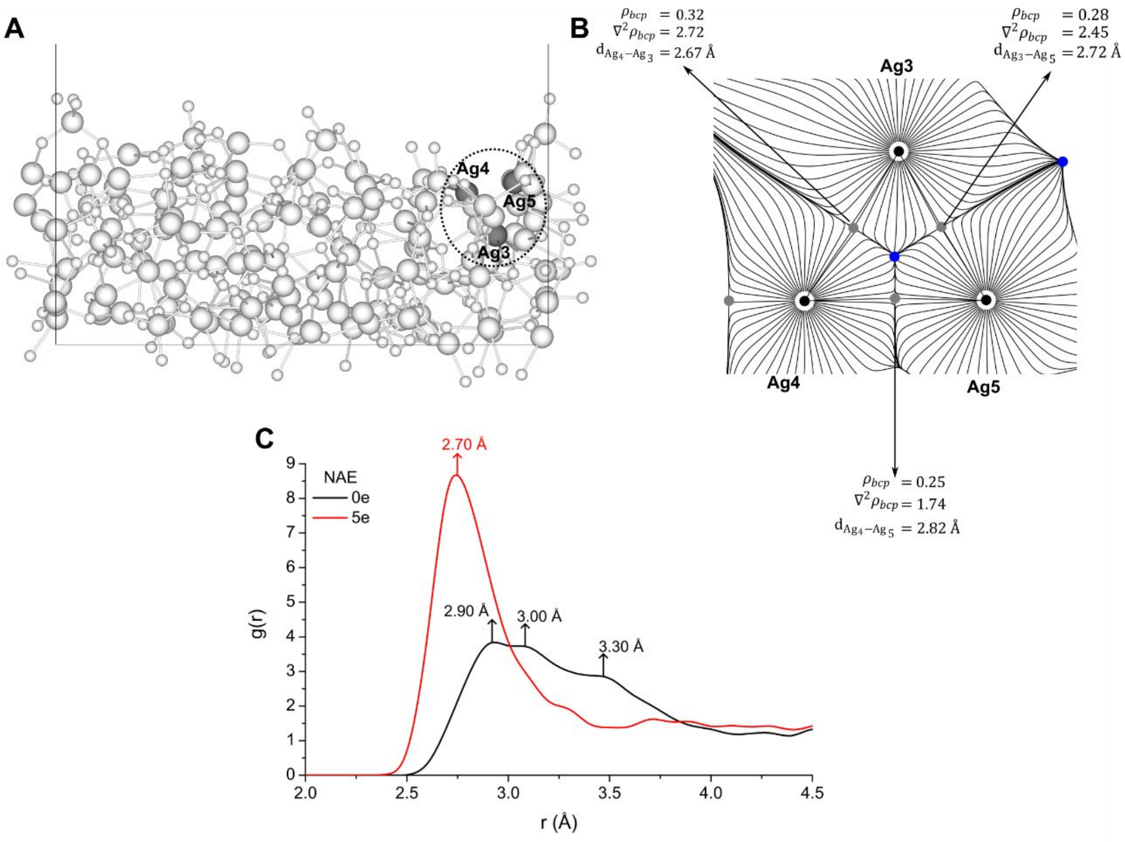

Figure S9. Representation of the clusters formed within the $(Ag_{96}W_{48}O_{192})$ model of the (011) surface when the NAE is 5e. In A, the trimer produced within the surface is illustrated. In B, the BCP (3, -1) of the Ag-Ag interactions within the cluster are shown, along with the distances between the Ag atoms, the charge density, and the charge density Laplacian. Panel C illustrates the pair correlation function for the Ag atoms.

Considering the Ag-Ag distances, we calculated the pair correlation function, g(r), for the (011) surface with a 2x2 model. It was evident that the most distinct peaks in the first coordination spheres are the same as in the surface with a 1x1 model. Additionally, with increasing NAE, the first peak increases intensity and shifts to shorter Ag-Ag distances while the other peaks disappear. This effect is associated with the tendency of the Ag atoms to maintain their local coordination, even with the intensification of the loss of structural order.

An analysis of the bond critical points BCP (3, -1) shows that the clusters produced within the surface are equivalent to those of the (011) surface with a 1x1 model. Furthermore, the trimer formed has distances shorter than those of FCC metallic silver (Figure S9) but of the same order as the smaller surface. Therefore, in the presence of a larger surface area and greater degrees of freedom, the electron absorption effect produced by the increase in NAE on the surface causes effects similar to those observed in the 1x1 model. For this reason, all subsequent studies were conducted on the smaller surface.

References

1. Wang, V., Xu, N., Liu, J.-C., Tang, G. & Geng, W.-T. VASPKIT: A user-friendly interface facilitating high-throughput computing and analysis using VASP code. Comput Phys Commun 267, 108033 (2021).
2. Róg, T., Murzyn, K., Hinsen, K. & Kneller, G. R. nMoldyn: A program package for a neutron scattering oriented analysis of molecular dynamics simulations. J Comput Chem 24, 657–667 (2003).
3. Kneller, G. R., Keiner, V., Kneller, M. & Schiller, M. nMOLDYN: A program package for a neutron scattering oriented analysis of Molecular Dynamics simulations. Comput Phys Commun 91, 191–214 (1995).
